# Optimal group and individual prenatal care visit patterns and preterm birth

**DOI:** 10.1186/s12884-025-07987-1

**Published:** 2025-08-14

**Authors:** Moonseong Heo, Jessica L. Britt, Lu Zhang, Emily A. Doherty, Amy H. Crockett

**Affiliations:** 1 Department of Public Health Sciences, College of Behavioral, Social and Health Sciences, Clemson University, 505 Edwards Hall, Clemson, SC 29634 USA; 2 Department of Obstetrics and Gynecology, Prisma Health, Greenville, SC USA; 3 Department of Rural Health, Oklahoma State University Center for Health Sciences, Tulsa, OK USA; 4 Division of Maternal Fetal Medicine Department of Obstetrics and Gynecology, University of South Carolina School of Medicine Greenville, Greenville, SC USA

**Keywords:** Preterm birth, Pregnant women group prenatal care, Individual prenatal care, Prenatal care patterns, Optimal prenatal care

## Abstract

**Background:**

Changes have recently been proposed to both the visit frequency and setting for delivery of prenatal care, including decreasing frequency of scheduled visits or using group visits or virtual visits. The impacts of participant engagement patterns with prenatal care on preterm birth (PTB) are not clearly understood. We aimed to characterize prenatal care visit patterns, examine their associations with PTB, and provide optimal cutoffs for care patterns.

**Methods:**

This study is a secondary analysis of prenatal care visit data obtained from the randomized CRADLE study that tested the effects of group (GPNC) versus individual (IPNC) prenatal care on PTB. We analyzed prenatal care visit data from *N* = 1,989 medically low-risk pregnant women who had at least one prenatal care visit between study enrollment and gestational age (GA) week 37. Prenatal care visit patterns before GA week 37 were predictors, characterized in terms of the number of IPNC and GPNC visits, duration of care, total hours of care, GA week at last visit, minimum gap (> 21 vs. ≤ 21 days) between any or GPNC visits, and discontinuation before the third trimester. PTB was the study outcome, defined as delivery < 37 GA weeks. Simple and multivariable logistic regression models and ROC analysis were applied to test associations and determine optimal cutoff points.

**Results:**

Overall, > 7 visits during pregnancy (OR = 0.58, 95%CI: 0.43–0.79, *p* < .001), > 3 visits in the third trimester (OR = 0.42, 95%CI: 0.30–0.57, *p* < .001), > 2.25 care hours during pregnancy (OR = 0.67, 95%CI: 0.49–0.92, *p* = .014), > 0.75 care hours during the third trimester (OR = 0.50, 95%CI: 0.37, 0.67, *p* < .001), and > 147 days in care (OR = 0.41, 95%CI: 0.30–0.56, *p* < .001) were all significantly associated with lower PTB rates. A minimum care gap of > 21 days was associated with higher PTB (aOR = 2.87, 95%CI: 1.76–4.69, *p* < .001) and discontinuation of care before the third trimester was the strongest correlate of PTB (aOR = 12.6, 95%CI: 6.5–24.5), *p* < .001) in terms of aOR compared to that of all the other pattern variables.

**Conclusion:**

Patient engagement with prenatal care providers, including both duration and frequency, was associated with reduced risk of PTB. Any proposed revision to the schedule or frequency of prenatal care which decreases patient contact with healthcare providers may risk worsening birth outcomes.

**Clinical trial registration:**

This study was registered on December 20, 2015, at ClinicalTrials.gov (www.clinicaltrials.gov, NCT02640638) with a title, A RCT of Centering Pregnancy on Birth Outcomes (CRADLE).

**Supplementary Information:**

The online version contains supplementary material available at 10.1186/s12884-025-07987-1.

## Introduction

The modern visit schedule and recommended content of prenatal care was developed in the 1930 s and has remained largely unchanged and unexamined in the decades since [1]. Recently, there have been new proposals for adapting both the structure of prenatal care visits to include both group visits as well as telehealth, as well as the frequency of care. Evaluation of the efficacy of these changes, particularly in terms of their impact on pregnancy outcomes, is limited. For example, given persistent high rates of poor birth outcomes, the prevention of PTB is a major goal of prenatal care [1, 2]. Preterm birth (PTB) is the second leading cause of newborn death and disability, and can lead to immediate and long-term health and developmental problems for the neonates [3, 4]. PTB occurs at high rates in the United States, where 10.2% of infants are born < 37 weeks gestational age (GA) in year 2022 [5]. The annual minimum total cost estimate for PTB is $26.2 billion dollars ($51,600 per child born prematurely), including medical care for the infant, maternal delivery costs, early intervention programs, special education for the four most common conditions associated with prematurity, and lost household productivity [6]. Clinical interventions to reduce PTB are included in the highest tier of priority topics for the Institute of Medicine research agenda [7]. 

The current standard individual prenatal care (IPNC) model includes approximately 13 visits scheduled with increasing frequency throughout pregnancy [1, 2, 8]. Patients who enter care late or attend fewer than the recommended number of visits have an increased risk of PTB, infant mortality, and preeclampsia [9–11]. However, IPNC visits are short, can feel hurried, and do not necessarily allow adequate time for healthcare providers to share all the anticipatory guidance pregnant individuals need and want [12, 13]. In contrast, the group prenatal care (GPNC) model bundles physical assessment with family and peer support and provider-led, patient-centered health education [14]. However, both a systematic review and our recent large-scale CRADLE randomized clinical trial revealed that IPNC and GPNC had comparable rates of PTB [15, 16]. 

There are, nonetheless, unanswered questions related to optimal care patterns and the mechanisms by which patient engagement with prenatal care providers are associated with adverse birth outcomes including PTB, and thus a more comprehensive investigation of the effect of patient engagement with prenatal care on PTB warranted. The present study sought to address this question by analyzing prenatal care visit data obtained from the CRADLE study by examining the impacts of patterns of participant engagement with IPNC and GPNC visits on PTB. Specifically, we aimed to characterize prenatal care visit patterns, examine their associations with PTB, and determine optimal prenatal care patterns.

## Methods

### Design and setting

This study is a secondary observational analysis of prenatal care visits data collected from the Centering Pregnancy on Birth Outcomes (CRADLE) study [16] which randomly assigned pregnant patients in a 1:1 allocation to GPNC or IPNC arm, stratified by self-reported race and ethnicity. The CRADLE study was designed to compare the effectiveness of GPNC versus IPNC prenatal care on PTB and low birth weight. The study recruited participants from a single practice site in Greenville, South Carolina, USA. Further details were reported elsewhere [17]. The study was approved by the Prisma Health Institutional Review Board (Pro00043994) and was registered at ClinicalTrials.gov (NCT02640638).

### Interventions

The IPNC arm provided participants with standard, traditional prenatal care in accordance with the schedule of visits recommended by the American College of Obstetricians and Gynecologists [1, 18]. Participants were scheduled to attend monthly provider visits for the first 28 weeks of pregnancy, every two to three weeks until 36 weeks, then weekly thereafter. Visits included an initial medical and psychosocial history, and ongoing physical assessment as well as patient education on pregnancy and prenatal care, options for intrapartum (delivery) care, and breastfeeding, and pediatrician selection.

For the GPNC arm, the CenteringPregnancy GPNC curriculum (Centering Healthcare Institute, Boston MA) for ten 2-hour GPNC sessions was and delivered in either English or Spanish to groups of 8–12 participants who were due to deliver in the same month. The CenteringPregnancy trademarked model includes a formal training and annual certification process to assure consistency in implementation [14, 19]. Participants received a patient workbook with health information and activities to promote goal setting and self-care, empowering them to become more engaged with and responsible for their health care. During the first 30 min, a healthcare provider conducts a brief physical assessment. The remaining 90 min are spent in a facilitated discussion in the group space. The curriculum includes family planning and childbirth preparation, stress management, newborn care, and parenting skills. GPNC participants were allowed to have additional IPNC visits outside of the 10 scheduled GPNC sessions if needed. In both groups, participants received routine screenings as well as specialized tests, interventions, and referrals depending on risk factors and the course of pregnancy.

### Outcome measure

The primary outcome for this secondary analysis was PTB defined as live delivery < 37 GA weeks. The best obstetrical estimate of GA was used in all cases, and all participants had ultrasound confirmation of pregnancy dating < 20 weeks of gestation as the standard of care [20, 21]. Delivery data were extracted from the electronic medical record (Epic Systems Incorporated, Verona, WI), and reviewed and validated by abstractors independent of the medical team.

### Other measures

Participants completed a survey instrument at enrollment, including but not limited to baseline socio-demographic factors (maternal age, race, employment, primary language, education, marital status, pregnancy intention, dental visits in the past 2 years, insurance, parity). From electronic health record were obtained maternal health behavior or clinical factors (during pregnancy: smoking, drinking, hypertension, any vaginal infection, any vaginal bleeding, and antepartum testing during pregnancy; and at enrollment: GA week, body mass index/weigh status, history of PTB experience).

### Participants

Inclusion criteria of the CRADLE study included medically low-risk pregnant women aged 14–45 years, singleton pregnancy, entry to care < 20 6/7 weeks GA, and GA < 24 0/7 weeks at enrollment. Exclusion criteria included medical or pregnancy complications, current incarceration and severe psychiatric illness that would preclude prenatal care delivery by a nurse practitioner or an indication for planned PTB. We did not exclude patients who were having active substance use disorder and/or on mediation-assisted-therapies as formal screening was not conducted. Self-reported rates of marijuana (5.0%) use in pregnancy were low in this cohort, and we believe that our rates of other substance use were similarly low in the overall study population. Experiences of domestic violence were evaluated separately by our social workers, but this was not known at the time of study recruitment thus such patients were not necessarily excluded. Although we screened the patients for psychiatric history especially on depression and anxiety as part of their general intake to prenatal care, we did not exclude patients based on these screenings. Participants could only participate in the study with one pregnancy to maintain independence. Participants were enrolled between February 2016 and March 2020 when continuation of in-person GPNC sessions became no longer safe due to the COVID-19 pandemic.

For the present study, we included participants who had a live birth and at least one completed initial, routine, or GPNC visits between study enrollment and GA week 36 6/7. We limited the GA week at 36 6/7 to make the number of visits and duration of care comparable between participants with full term and preterm birth, for participants with preterm birth by definition do not have any prenatal care visits after GA week 37.

### Characterizations of care visit patterns

To characterize visit patterns which will serve as the predictors, or exposure variables, for the PTB, we included the following visit types with completed in-person appointments, made between study enrollment and GA week 36 6/7: initial IPNC (1 h; establishment of prenatal care), return IPNC (1/4 hours; after the initial prenatal care visit), and GPNC (2 h) visits for the GPNC arm only. Both groups received an initial IPNC visit at intake to care in order to ensure they met practice guidelines for eligibility for GPNC. As previously stated, the IPNC arm received only return prenatal visits for the duration of their prenatal care while the GPNC were allowed to supplement with return IPNC visits as necessary in addition to their GPNC visits. Visit pattern variables were as follows: (1) total number of any visits (regardless of visit types), (2) total number of any visits in each trimester (first trimester: GA ≤ 12 weeks; second trimester: 12 < GA ≤ 27; third trimester: GA > 27 weeks), (3) total care hours defined by the above estimated timeframes for each visit type, (4) total care hours in each trimester; (5) duration of care defined as the length in days from the first to the last prenatal care visit, (6) dichotomized minimum care gap (≤ 21 and > 21 days (or 3 weeks), determined through empirical explorations) between any visits, (7) GA at the last visit, and (8) discontinuation of care before the third trimester (or before GA 27), i.e., no visits in the third trimester. In addition, we also characterize the following pattern variables only for participants in the GPNC arm: (1) total number of GPNC visits, (2) number of GPNC visits in each trimester, (3) dichotomized minimum care gap (≤ 21 and > 21 days, determined based on empirical explorations) between GPNC visits. Of note, when a participant only made a single visit during the study period, then the care gap was calculated as days between that single visit date and delivery date or GA week 37 date whichever is earlier. We also did not count visits, care hours, care gap, care length in days after GA week 37 for participants with a full-term birth.

### Statistical analysis

Descriptive statistics included frequency, percentages, mean and standard deviations (SDs). Baseline socio-demographic, health behavior and clinical factors were compared between participants with and without PTB using t-tests and Chi-square tests, and so were care visit pattern variables. To examine associations between pattern variables and PTB, we applied simple and multivariable logistic regressions to estimate unadjusted crude odds-ratios (ORs) and adjusted odds-ratios (aORs) along with 95%CIs, respectively. For the multivariable logistic regressions, we included for adjusting purposes the characteristic variables that were significantly different between PTB and non-PTB participants, which included history of PTB, hypertension, any vaginal bleeding, and antepartum testing. In addition, we also included GA at enrollment as it may influence the pattern variables through duration of care. For instance, an earlier GA at enrollment could be associated with a greater opportunity for a greater number of visits. Once significant pattern variables were identified, we determined optimal cutoff points of those variables based on receiver operating curve (ROC) analysis results including Youden index and reported area under curve (AUC), sensitivity, specificity, positive predictive value (PPV), and negative predictive values (NPV). From thereof, we estimated crude ORs for PTB along with 95% CIs between participants with lower and higher cutoff values of pattern variables. All analyses were conducted separately for all, GPNC, and IPNC participants using SAS v9.4 (SAS Inc., Cary, NC). Statistical significance was declared when a two-sided p-value was less than 0.05.

## Results

### Participants and characteristics at baseline and during pregnancy

Out of 2,350 randomized participants enrolled in the CRADLE study, the present study included 1,989 (985 for GPNC and 1,004 for IPNC group) participants who had at least one completed GPNC or IPNC care visit during the aforementioned time period for this study. The total number of visits was 17,882 with 1,474 initial care visits, 11,768 routine individual care visits and 4,640 GPNC visits between study enrollment and GA 36 6/7. Descriptive statistics for characteristics at baseline and during pregnancy are presented in Table 1. Briefly, the mean (SD) baseline maternal age was 25.2(5.4) years and 12.3(3.9) weeks for the baseline GA week, which was not significantly different between the two arms. The median (q1, q3) for the PTB delivery GA was 35.6 (33.7, 36.3) weeks. No baseline socio-demographic factors were significantly different between participants with and without PTB, nor were any behavior factors during pregnancy. However, clinical factors including history of PTB, hypertension, any vaginal bleeding and antepartum testing were all significantly greater for PTB.

**Table 1:** Comparisons of characteristics between patients with and without preterm birth

		Preterm Birth: Mean (SD), *N*(%^a^)		
Characteristics	All: Mean (SD), *N* (%^b^)	Yes (*N* = 187, 9.4%)	No (*N* = 1,802, 90.6%)	*p* ^c^
**Socio-demographic factors at enrollment**				
Maternal age, years	25.2 (5.4)	25.9 (5.6)	25.1 (5.4)	0.058
Race				0.142
African American	783 (39.4%)	85 (10.8%)	698 (89.1%)	
Latina	457 (23.0%)	38 (8.3%)	419 (91.7%)	
White	724 (36.3%)	64 (8.8%)	660 (91.2%)	
Other	25 (1.3%)	0 (0.0%)	25 (100.0%)	
Employment				0.789
Full or part time	988 (53.2%)	92 (9.3%)	896 (90.7%)	
Other	868 (46.8%)	84 (9.7%)	784 (90.3%)	
English as the primary language				0.551
Yes	1,642 (82.9%)	158 (9.6%)	1,484 (90.4%)	
No	338 (17.1%)	29 (8.6%)	309 (91.4%)	
Education				0.824
Lower than high school	513 (27.3%)	49 (9.6%)	464 (90.5%)	
High school or higher	1,367 (72.7%)	126 (9.2%)	1,241 (90.8%)	
Marital Status				0.866
Married	426 (24.0%)	29 (6.8%)	397 (93.2%)	
Other	1,348 (76.0%)	95 (7.0%)	1,253 (93.0%)	
Pregnancy Intention				0.192
Yes	639 (32.1%)	68 (10.6%)	571 (89.4%)	
No	1,350 (67.9%)	119 (8.8%)	1,231 (91.2%)	
Dental visits within the past 2 years				0.668
Yes	1,161 (60.2%)	105 (9.0%)	1,056 (91.0%)	
No	769 (39.8%)	74 (9.6%)	695 (90.4%)	
Insurance any time within the past year				0.608
Yes	958 (49.6%)	94 (9.8%)	864 (90.2%)	
No	975 (50.4%)	89 (9.1%)	886 (90.9%)	
**Behavioral factors during pregnancy**				
Smoking				0.720
Yes	354 (18.3%)	31 (8.8%)	323 (91.2%)	
No	1,580 (81.7%)	148 (9.4%)	1,432 (90.6%)	
Drinking				0.804
Yes	71 (3.7%)	6 (8.5%)	65 (91.6%)	
No	1,867 (96.3%)	174 (9.3%)	1,693 (90.7%)	
**Clinical factors at enrollment**				
Gestational age week	12.3 (3.9)	12.3 (4.2)	12.3 (3.9)	0.994
Parity				0.595
Nulliparous	857 (43.1%)	84 (9.8%)	773 (90.2%)	
Multiparous	1,137 (56.9%)	103 (9.1%)	1,029 (90.9%)	
Body mass index	28.9 (7.2)	29.5 (7.9)	28.9 (7.1)	0.242
Weight status				0.560
Underweight	64 (3.2%)	7 (10.9%)	57 (89.1%)	
Normal	638 (32.1%)	58 (9.1%)	580 (90.9%)	
Overweight	504 (25.4%)	41 (8.1%)	463 (91.9%)	
Obese	781 (39.3%)	81 (10.4%)	700 (89.6%)	
History of preterm birth				**< 0.001**
Yes	192 (9.6%)	42 (21.9%)	150 (78.1%)	
No	1,797 (90.4%)	145 (8.6%)	1,652 (91.9%)	
**Clinical factors during pregnancy**				
Hypertension				**< 0.001**
Yes	712 (35.8%)	100 (14.0%)	612 (86%)	
No	1,277 (64.2%)	87 (6.8%)	1,190 (93.2%)	
Any vaginal infection				0.948
Yes	685 (34,4%)	64 (9.3%)	621 (90.7%)	
No	1,304 (65.6%)	123 (9.4%)	1,181 (90.6%)	
Any vaginal bleeding				**0.020**
Yes	141 (7.1%)	21 (14.9%)	120 (85.1%)	
No	1,848 (92.9%)	166 (9.0%)	1,682 (91.0%)	
Antepartum testing				**< 0.001**
Yes	552 (27.7%)	94 (17.0%)	458 (82.9%)	
No	1,437 (72.3%)	93 (6.5%)	1,344 (93.5%)	

^a^Row percentages; ^b^Column percentages; ^c^p-values were obtained based on two-sample t-tests or Chi-square tests

### Comparisons of pattern variables

Descriptive statistics for pattern variables are presented in Table 2. Overall, the mean (SD) was 34.1(2.7) for GA week at the last visit, 127 (6.4%) had longer than 21 days for minimum care gap, and 44 (2.7%) participants discontinued visits/care before the third trimester. The GPNC arm had 4.4 (3.1) centering visits on average, 135 of 773 (17.5%) had longer than 21 days for minimum GPNC visit gap days, and 212 participants did not have any GPNC visits. Compared with the IPNC arm, the GPNC arm had significantly earlier last visit (33.9 (2.8) vs. 34.3 (2.6), *p* =.008) GA weeks, greater total number of any visits (8.0 (2.6) vs. 7.7 (2.3), *p* <.001), especially during the second trimester (3.7 (1.4) vs. 3.3 (1.2), *p* <.001), and longer overall total care hours (10.2 (5.8) vs. 2.6 (1.4), *p* <.001) as well as in each trimester.

**Table 2:** Distribution of care pattern variables assessed between study enrollment and gestational age week 36 6/7

	Mean (SD), %			
Pattern Variables	All (*N* = 1,989)	IPNC arm (*N* = 1,004)	GPNC arm (*N* = 985)	*p*
Gestational Age Week at last visit	34.1 (2.7)	34.3 (2.6)	33.9 (2.8)	**0.008**
Total number of any visits	7.8 (2.5)	7.7 (2.3)	8.0 (2.6)	**< 0.001**
Number of any visits in the first TM	0.7 (0.7)	0.7 (0.7)	0.7 (0.7)	0.412
Number of any visits in the second TM	3.5 (1.3)	3.3 (1.2)	3.7 (1.4)	**< 0.001**
Number of any visits in the third TM	3.6 (1.6)	3.6 (1.6)	3.6 (1.7)	0.883
Total number of GPNC visits	NA	NA	4.4 (3.1)	NA
Number of GPNC visits in the first TM	NA	NA	0.1 (0.2)	NA
Number of GPNC visits in the second TM	NA	NA	2.2 (1.6)	NA
Number of GPNC visits in the third TM	NA	NA	2.1 (1.7)	NA
Total care hours during pregnancy	6.4 (5.7)	2.6 (1.4)	10.2 (5.8)	**< 0.001**
Total care hours in the first TM	0.6 (0.7)	0.6 (0.5)	0.7 (0.8)	**< 0.001**
Total care hours in the second TM	3.0 (2.9)	1.1 (0.8)	5.0 (3.0)	**< 0.001**
Total care hours in the third TM	2.8 (3.0)	1.0 (0.7)	4.6 (3.3)	**< 0.001**
Duration of care in days	148.7 (35.9)	149.2 (36.7)	148.2 (35.2)	0.542
Minimum gap between any visits > 21 days	127 (6.4%)	74 (7.4%)	53 (5.4%)	0.070
Minimum gap between GPNC visits > 21 days	NA	NA	135/773^a^ (17.5%)	NA
Discontinuation of care before the third TM	44 (2.7%)	19 (1.9%)	25 (2.5%)	0.328

*Abbreviations*; *IPNC *Individual prenatal care, *GPNC *Groups prenatal care, *TM *Trimester, *NA *Not applicable. ^a^*N*=212 had no GPNC visits

### Associations between pattern variables and PTB

The overall PTB rate was 9.4% (Table 1) and adjusted ORs of pattern variables with PTB stratified by the study groups are displayed in Table 3. Regardless of the study arm, a later visit date before week 36 6/7, greater number of visits, longer total care hour and, longer duration of care, were all associated with lower PTB rate. In particular, greater number of visits and total care hours during the third trimester were both significantly associated with lower PTB rate. Similarly, in the GPNC group, greater number of GPNC visits was associated with lower PTB rate. A minimum care gap longer than 21 days was significantly associated with higher rate of PTB in both arms, and when combined as well (aOR = 2.87, 95%CI: 1.76–4.69, *p* <.001). Notably, discontinuation of care before the third trimester was significantly associated with higher PTB rate among all (aOR = 12.6, 95%CI: 6.5–24.5), *p* <.001), IPNC arm (aOR = 23.0, 95%CI: 8.1–65.3), *p* <.001), and GPNC arm (aOR = 10.1, 95%CI: 4.1–25.1), *p* <.001) participants. Crude unadjusted ORs are presented in Supplementary Table 1, which shows little changes in estimates and p-values except that in the GPNC group the total number of visits was not significantly associated with PTB and minimum centering care gap > 21 days was significantly associated with higher PTB (crude OR = 2.06, 95%CI: 1.21–3.52, *p* =.008).

**Table 3:** Adjusted association between care pattern variables and preterm birth assessed between study enrollment and gestational age week 36 6/7

	All		IPNC arm		GPNC arm	
Pattern Variables	aOR (95%CI)	*p*	aOR (95%CI)	*p*	aOR (95%CI)	*p*
Gestational Age Week at last visit	0.78 (0.74, 0.82)	**< 0.001**	0.78 (0.72, 0.83)	**< 0.001**	0.78 (0.73, 0.83)	**< 0.001**
Total number of any visits	0.84 (0.78, 0.90)	**< 0.001**	0.79 (0.70, 0.88)	**< 0.001**	0.87 (0.79, 0.95)	**0.001**
Number of any visits in the first TM	1.05 (0.78, 1.42)	0.754	1.22 (0.76, 1.97)	0.415	0.98 (0.67, 1.44)	0.921
Number of any visits in the second TM	0.98 (0.87, 1.11)	0.800	0.82 (0.66, 1.02)	0.082	1.06 (0.91, 1.23)	0.459
Number of any visits in the third TM	0.70 (0.63, 0.77)	**< 0.001**	0.70 (0.60, 0.82)	**< 0.001**	0.69 (0.60, 0.80)	**< 0.001**
Total number of GPNC visits	NA	NA	NA	NA	0.91 (0.85, 0.98)	**0.009**
Number of GPNC visits in the first TM	NA	NA	NA	NA	0.26 (0.06, 1.12)	0.071
Number of GPNC visits in the second TM	NA	NA	NA	NA	0.95 (0.83, 1.08)	0.423
Number of GPNC visits in the third TM	NA	NA	NA	NA	0.78 (0.69, 0.89)	**< 0.001**
Total care hours during pregnancy	0.98 (0.95, 1.01)	0.262	0.58 (0.40, 0.84)	**0.004**	0.95 (0.91, 0.99)	**0.006**
Total care hours in the first TM	0.98 (0.71, 1.35)	0.894	1.32 (0.67, 2.59)	0.422	0.86 (0.59, 1.24)	0.413
Total care hours in the second TM	1.02 (0.97, 1.08)	0.406	0.72 (0.44, 1.16)	0.176	0.98 (0.91, 1.05)	0.563
Total care hours in the third TM	0.91 (0.86, 0.97)	**0.004**	0.28 (0.15, 0.52)	**< 0.001**	0.86 (0.80, 0.93)	**< 0.001**
Duration of care in days	0.98 (0.97, 0.98)	**< 0.001**	0.98 (0.97, 0.98)	**< 0.001**	0.98 (0.97, 0.98)	**< 0.001**
Minimum gap between any visits > 21 days	2.87 (1.76, 4.69)	**< 0.001**	4.07 (2.10, 7.89)	**< 0.001**	2.29 (1.06, 4.92)	**0.034**
Minimum gap between GPNC visits > 21 days	NA	NA	NA	NA	1.72 (0.98, 3.02)	0.060
Discontinuation of care before the third TM	12.6 (6.5, 24.5)	**< 0.001**	23.0 (8.1, 65.3)	**< 0.001**	10.1 (4.1, 25.1)	**< 0.001**

*Abbreviations*; *IPNC *Individual prenatal care, *GPNC *Groups prenatal care, *TM *Trimester, *NA *Not applicable, *aOR *Adjusted odds-ratio, *CI *Confidence interval

### Optimal cutoff points of pattern variables

The ROC curve analysis results in terms of cutoff points or optimal threshold levels, AUC, sensitivity, specificity, PPV, NPV and Youden index are presented in Supplementary Table 2 stratified by the study groups. Among IPNC participants, > 6 visits during pregnancy, > 3 visits in the trimester, > 2.25 care hours during pregnancy, > 0.75 care hours during the third trimester, and > 142 day duration of care were all significantly associated with lower PTB rates (Table 4). Among the GPNC arm participants, > 5 GPNC visits during pregnancy, > 2 centering visits during the third trimester, > 11.75 total care hours during pregnancy, > 4.75 total care hours during the third trimester, and > 154 day duration of care were all significantly associated with lower PTB rate. When both groups are combined, participants with > 7 visits during pregnancy (OR = 0.58, 95%CI: 0.43–0.79, *p* <.001), > 3 visits in the trimester (OR = 0.42, 95%CI: 0.30–0.57, *p* <.001), > 2.25 care hours during pregnancy (OR = 0.67, 95%CI: 0.49–0.92, *p* =.014), > 0.75 care hours during the third trimester (OR = 0.50, 95%CI: 0.37, 0.67, *p* <.001), and > 147 day duration of care (OR = 0.41, 95%CI: 0.30–0.56, *p* <.001) were all significantly associated with lower PTB rates. (Table 4)

**Table 4:** Comparisons of PTB between below and above optimal threshold level of pattern variables assessed between study enrollment and gestational age week 36 6/7

Pattern Variable	Cut points	PTB: *n*/*N* (%)	OR (95%CI)	*p*
**ALL**				
Total number of any visits	> 7	79/1082 (7.3%)	0.58 (0.43, 0.79)	**< 0.001**
	≤ 7	108/907 (11.9%)		
Number of any visits in the third TM	> 3	62/1043 (5.9%)	0.42 (0.30, 0.57)	**< 0.001**
	≤ 3	125/946 (13.2%)		
Total care hours during pregnancy	> 2.25	120/1430 (8.4%)	0.67 (0.49, 0.92)	**0.014**
	≤ 2.25	67/559 (12.0%)		
Total care hours in the third TM	> 0.75	96/1321 (7.3%)	0.50 (0.37, 0.67)	**< 0.001**
	≤ 0.75	91/668 (13.6%)		
Duration of care in days	> 147	76/1201 (6.3%)	0.41 (0.30, 0.56)	**< 0.001**
	≤ 147	111/788 (14.1%)		
**IPNC arm**				
Total number of any visits	> 6	43/693 (6.2%)	0.45 (0.28, 0.71)	**< 0.001**
	≤ 6	40/311 (12.9%)		
Number of any visits in the third TM	> 3	29/538 (5.4%)	0.43 (0.27, 0.70)	**< 0.001**
	≤ 3	54/466 (11.6%)		
Total care hours during pregnancy	> 2.25	37/588 (6.3%)	0.54 (0.34, 0.85)	**0.007**
	≤ 2.25	46/416 (11.1%)		
Total care hours in the third TM	> 0.75	30/546 (5.5%)	0.44 (0.28, 0.71)	**< 0.001**
	≤ 0.75	53/458 (11.6%)		
Duration of care in days	> 142	32/646 (5.0%)	0.31 (0.20, 0.50)	**< 0.001**
	≤ 142	51/358 (14.2%)		
**GPNC arm**				
Total number of any visits	> 7	48/567 (8.5%)	0.60 (0.40, 0.90)	**0.013**
	≤ 7	56/418 (13.4%)		
Number of any visits in the third TM	> 3	33/505 (6.5%)	0.40 (0.26, 0.62)	**< 0.001**
	≤ 3	71/480 (14.8%)		
Total number of GPNC visits	> 5	30/440 (6.8%)	0.47 (0.30, 0.73)	**< 0.001**
	≤ 5	74/545 (13.6%)		
Number of GPNC visits in the third TM	> 2	26/456 (5.7%)	0.35 (0.22, 0.56)	**< 0.001**
	≤ 2	78/529 (14.7%)		
Total care hours during pregnancy	> 11.75	31/451 (6.9%)	0.47 (0.30, 0.72)	**< 0.001**
	≤ 11.75	73/534 (13.7%)		
Total care hours in the third TM	> 4.75	26/460 (5.7%)	0.34 (0.22, 0.55)	**< 0.001**
	≤ 4.75	78/525 (14.9%)		
Duration of care in days	> 154	35/498 (7.0%)	0.46 (0.30, 0.70)	**< 0.001**
	≤ 154	69/487 (14.2%)		

*Abbreviations*; *PTB *Preterm birth, *IPNC *Individual prenatal care, *GPNC *Groups prenatal care, *TM *Trimester, *OR *Odds-ratio, *CI *Confidence interval

## Discussion

The present study provides rigorous data-driven prenatal care pattern cutoff points for reduced risk of PTB in terms of care length, routine or GPNC visits, and the gap between visits. Briefly, longer care hours and duration of care, more visits (either IPNC or GPNC) and a shorter gap in care were all associated with lower risk of PTB. In particular, discontinuation of care visits before the third trimester is most strongly associated with increased risk of PTB. Consistent with this finding, care in the third trimester seemed to be particularly critical since both increased visits and care hours during the third trimester were associated with reduced risk of PTB, but this same benefit was not seen in the first or second trimester.

The association between suboptimal care based on cutoffs of our care pattern variables and higher rate of PTB may be due in part to the missed opportunity for timely detection of risk factors through ultrasound screening, or other means. If the risk of PTB had been detected at an earlier stage of pregnancy, for example, then patients who ended up with PTB could have had more frequent prenatal care visits. However, this kind of potential confounding by indication, meaning association between greater number of visits and higher rate of PTB, was not observed in our study. At the same time, it is unlikely that the association between suboptimal care and PTB might be due to reverse causation, since the timing of the first visit was adjusted and a majority of PTB patients delivered after 35.6 GA weeks. Therefore, it might be critical to identify obstacles/barriers to or determinants of optimal care [22–24], that are pertinent to practice settings and patient characteristics such as cultural, race and ethnicity, socioeconomic, social determinants of health, and clinical backgrounds. In addition, more intensive monitoring by increasing touch points including phone calls, text messaging, apps, or even home visiting [25] might warrant future research to reduce PTB risk.

Other studies have sought to define the optimal number of prenatal care visits in order to optimize pregnancy outcomes. The most widely used measure in public health, including the adequacy of prenatal care utilization index initially published by Kotelchuck [26], utilize visit frequency and timing of entry to prenatal care [27, 28]. More recently, authors have recommended a reduced number of prenatal care visits is not associated with increased risk of PTB among low-risk pregnant women [29–34]. However, much of the research suggesting equivalence between reduced and standard care schedules took place in the late 1990 s and early 2000 s [33, 34]. Although Carter et al. [32] found no difference in a composite measure of adverse neonatal outcomes between patients with ≤ 10 and those with > 10 prenatal care visits, but patients with PTB were excluded from analysis. Our study, however, includes not only the frequency of patient engagement with prenatal care, but also explores the influence of the type or modality of prenatal care. This is particularly relevant as recently there have been efforts to offer patients more tailored prenatal care experiences, including GPNC or telehealth visits to better meet their needs and preferences for care although a growing body of evidence shows no difference in rates of PTB for women in GPNC compared to IPNC [15, 16, 35–38]. 

A more recent study found a technology enhanced care model incorporating virtual visits, home monitoring devices, and a nurse moderated online community was associated with higher satisfaction and lower stress relative to a standard visit schedule, however the study was underpowered to detect differences in neonatal outcomes such as preterm birth [29]. Technology-based enhancements were not considered in the present study; therefore, it is possible that the observed benefits of additional visits and time in care for reduced PTB could be lessened through ancillary supports such as tele-visits and at home monitoring devices. Nevertheless, there is low strength evidence at present that rates of PTB do not differ between hybrid and standard in-person care models, and therefore more research is needed particularly among subgroups, as there is concern that telehealth may reinforce health inequities [39].

### Study strengths

The parent CRADLE study is a large randomized clinical trial that compared IPNC and GPNC which recruited a diverse study population with minimized lost follow-up evaluations for outcomes which were rigorously validated by clinical team experienced with CenteringPregnancy GPNC model predating the clinical trial by many years. To our knowledge, the present study is the first to suggest characterizations of prenatal care patterns and examine their associations with PTB outcomes along with optimal cutoffs.

### Study Limitations 

As a secondary analysis, the current study shares the limitations of the parent CRADLE study, which was conducted at a single study site that generally serves a low-income population. Additionally, patients who entered prenatal care late or had pre-existing medical complications were excluded, which limits the generalizability of the present study findings. Furthermore, the secondary analysis sample may not necessarily be a representative sample of the parent CRADLE population due to the exclusion of patients with no visit data. Prenatal care visits before study enrollment were not considered although based on recruitment procedures, it is unlikely that more than one visit would have been missed. Although only visits before GA week 36 6/7 were considered and the first visit GA week was controlled in the analysis, this still may not completely adjust for *potential* care patterns of participants with earlier PTB that would have been observed without PTB. It presents an interesting epidemiology methodological question as to how to more properly adjust for analysis such truncations of pattern variable values imposed by shortened GA weeks due to PTB in a causal inference manner as this study was not able to completely tease out cause and effect between care patterns and PTB.

### Future studies

Built upon findings from this study, the following future studies might be worth considering. First, to examine the effect of care visit or provider encounter patterns specifically targeting spontaneous PTB and to compare that between GPNC and IPNC among patients with prior history of PTB given increased risk of PTB among these individuals. Second, to identify factors associated with parental care visit patterns such as larger gaps or early discontinuation that are not necessarily directly impacted by delivery timing as the populations with such patterns would benefit most from interventions to encourage prenatal care. Third, to examine the effect of a reallocation of visit frequency as to whether for instance visits during the second trimester be reduced in favor of intensified monitoring during the third trimester. Fourth, to examine the impact of visit frequency on other birth outcomes like hypertensive disorders of pregnancy, maternal mental health outcomes like postpartum depression, or health behaviors like breastfeeding initiation.

## Conclusion

This secondary analysis should serve as an evidence-based approach for optimizing the number and frequency of prenatal care visits, and our inclusion of two different care models provides insight into the importance of participant engagement, and the potential for improving PTB outcome by increasing the number of care visits, care hours, duration of care, and reducing visit gaps. Receiving cares during the third trimester was more important than during the other trimesters, regardless of timing of entry to care. Prenatal care models which reduce the number of visits or otherwise decrease patient engagement with healthcare providers may risk adverse birth outcomes and thus should be carefully reevaluated.

## Supplementary Information

Supplementary Material 1.

## Data Availability

All data generated or analyzed during this study are included in this published article and its supplementary information file.

## Abbreviations

AUC: Area Under Curve
aOR: Adjusted Odds-Ratio
CI: Confidence Interval
COVID-19: Coronavirus Disease 2019
GA: Gestational Age
GPNC: Group Prenatal Care
IPNC: Individual Prenatal Care
MA: Massachusetts
NA: Not Applicable
NC: North Carolina
NPV: Negative Predictive Value
OR: Odds-Ratio
PPV: Positive Predictive Value
PTB: Preterm Birth
ROC: Receiver Operating Curve
SAS: Statistical Analysis System
TM: Trimester
USA: United States of America
