# Supplementary material for: Optimal group and individual prenatal care visit patterns and preterm birth
Source: BMC Pregnancy Childbirth. 2025 Aug 14;25:848. doi: 10.1186/s12884-025-07987-1 (PMC12351913; doi:10.1186/s12884-025-07987-1)
Supplement: Supplementary file 1 — Supplementary Material 1. [file 12884_2025_7987_MOESM1_ESM.docx]

**Supplementary Table 1: Unadjusted association between care pattern variables and preterm birth**

|  | **All** | | **IPNC arm** | | **GPNC arm** | |
| --- | --- | --- | --- | --- | --- | --- |
| **Pattern Variables** | **OR (95%CI)** | **p** | **OR (95%CI)** | **p** | **OR (95%CI)** | **p** |
| Gestational Age Week at last visit | 0.79 (0.76, 0.83) | **<.001** | 0.80 (0.74, 0.85) | **<.001** | 0.80 (0.75, 0.85) | **<.001** |
| Total number of any visits | 0.90 (0.84, 0.95) | **<.001** | 0.83 (0.75, 0.92) | **<.001** | 0.93 (0.86, 1.01) | 0.085 |
| Number of any visits in the first TM | 1.08 (0.88, 1.33) | 0.462 | 0.96 (0.70, 1.32) | 0.802 | 1.17 (0.89, 1.53) | 0.254 |
| Number of any visits in the second TM | 1.05 (0.94, 1.18) | 0.379 | 0.87 (0.71, 1.05) | 0.149 | 1.15 (0.99, 1.32) | 0.061 |
| Number of any visits in the third TM | 0.73 (0.66, 0.80) | **<.001** | 0.73 (0.63, 0.85) | **<.001** | 0.72 (0.63, 0.83) | **<.001** |
| Total number of GPNC visits | NA | NA | NA | NA | 0.90 (0.84, 0.96) | **0.002** |
| Number of GPNC visits in the first TM | NA | NA | NA | NA | 0.29 (0.07, 1.20) | 0.088 |
| Number of GPNC visits in the second TM | NA | NA | NA | NA | 0.94 (0.83, 1.07) | 0.333 |
| Number of GPNC visits in the third TM | NA | NA | NA | NA | 0.76 (0.67, 0.86) | **<.001** |
| Total care hours during pregnancy | 0.98 (0.95, 1.01) | 0.148 | 0.67 (0.49, 0.90) | **0.008** | 0.95 (0.91, 0.98) | **0.003** |
| Total care hours in the first TM | 1.01 (0.80, 1.27) | 0.936 | 0.93 (0.61, 1.43) | 0.752 | 1.02 (0.78, 1.33) | 0.888 |
| Total care hours in the second TM | 1.01 (0.96, 1.06) | 0.662 | 0.77 (0.49, 1.20) | 0.248 | 0.98 (0.91, 1.04) | 0.485 |
| Total care hours in the third TM | 0.91 (0.86, 0.96) | **0.002** | 0.33 (0.18, 0.60) | **<.001** | 0.85 (0.80, 0.91) | **<.001** |
| Duration of care in days | 0.99 (0.99, 0.99) | **<.001** | 0.99 (0.98, 0.99) | **<.001** | 0.99 (0.99, 1.00) | **<.001** |
| Minimum gap between any visits >21 days | 2.57 (1.61, 4.10) | **<.001** | 3.22 (1.74, 5.98) | **<.001** | 2.07 (1.01, 4.26) | **0.047** |
| Minimum gap between GPNC visits >21 days | NA | NA | NA | NA | 2.06 (1.21, 3.52) | **0.008** |
| Discontinuation of care before the third TM | 8.9 (4.8, 16.4) | **<.001** | 13.9 (5.5, 35.2) | **<.001** | 6.1 (2.7, 14.1) | **<.001** |

**Supplementary Table 2: Optimal cut points predicting PTB determined based on Youden index**

| **Pattern Variables** | **AUC (95%CI)** | **Cut point** | **Sensitivity** | **Specificity** | **PPV** | **NPV** | **Youden Index** |
| --- | --- | --- | --- | --- | --- | --- | --- |
| ALL |  |  |  |  |  |  |  |
| Total number of any visits | 0.59 (0.54, 0.63) | ≤ 7 | 0.58 | 0.56 | 0.12 | 0.93 | 0.13 |
| Number of any visits in the third TM | 0.64 (0.59, 0.68) | ≤ 3 | 0.67 | 0.54 | 0.13 | 0.94 | 0.21 |
| Total care hours during pregnancy | 0.54 (0.50, 0.59) | ≤ 2.25 | 0.36 | 0.73 | 0.12 | 0.92 | 0.09 |
| Total care hours in the third TM | 0.60 (0.55, 0.64) | ≤ 0.75 | 0.49 | 0.68 | 0.14 | 0.93 | 0.17 |
| Duration of care in days | 0.63 (0.59, 0.67) | ≤147 | 0.59 | 0.62 | 0.14 | 0.94 | 0.22 |
| IPNC |  |  |  |  |  |  |  |
| Total number of any visits | 0.62 (0.55, 0.68) | ≤ 6 | 0.48 | 0.71 | 0.13 | 0.94 | 0.19 |
| Number of any visits in the third TM | 0.62 (0.55, 0.69) | ≤ 3 | 0.65 | 0.55 | 0.12 | 0.95 | 0.20 |
| Total care hours during pregnancy | 0.59 (0.52, 0.66) | ≤ 2.25 | 0.55 | 0.60 | 0.11 | 0.94 | 0.15 |
| Total care hours in the third TM | 0.61 (0.55, 0.68) | ≤ 0.75 | 0.64 | 0.56 | 0.12 | 0.95 | 0.20 |
| Duration of care in days | 0.65 (0.59, 0.72) | ≤ 142 | 0.61 | 0.67 | 0.14 | 0.95 | 0.28 |
| GPNC |  |  |  |  |  |  |  |
| Total number of any visits | 0.57 (0.51, 0.63) | ≤ 7 | 0.54 | 0.59 | 0.13 | 0.92 | 0.13 |
| Number of any visits in the third TM | 0.65 (0.59, 0.71) | ≤ 3 | 0.68 | 0.54 | 0.15 | 0.93 | 0.22 |
| Total number of GPNC visits | 0.60 (0.55, 0.65) | ≤ 5 | 0.71 | 0.47 | 0.14 | 0.93 | 0.18 |
| Number of GPNC visits in the third TM | 0.63 (0.58, 0.68) | ≤ 2 | 0.75 | 0.49 | 0.15 | 0.94 | 0.24 |
| Total care hours during pregnancy | 0.59 (0.54, 0.65) | ≤ 11.75 | 0.70 | 0.48 | 0.14 | 0.93 | 0.18 |
| Total care hours in the third TM | 0.65 (0.59, 0.70) | ≤ 4.75 | 0.75 | 0.49 | 0.15 | 0.94 | 0.24 |
| Duration of care in days | 0.61 (0.56, 0.67) | ≤ 154 | 0.66 | 0.53 | 0.14 | 0.93 | 0.19 |

Abbreviations; IPNC: individual prenatal care; GPNC: groups prenatal care; TM: trimester; AUC: Area under curve; PPV: Positive predictive value; NPV: predictive negative value
